# Structure-Bioactivity Relationship for Benzimidazole Thiophene Inhibitors of Polo-Like Kinase 1 (PLK1), a Potential Drug Target in *Schistosoma mansoni*

**DOI:** 10.1371/journal.pntd.0004356

**Published:** 2016-01-11

**Authors:** Thavy Long, R. Jeffrey Neitz, Rachel Beasley, Chakrapani Kalyanaraman, Brian M. Suzuki, Matthew P. Jacobson, Colette Dissous, James H. McKerrow, David H. Drewry, William J. Zuercher, Rahul Singh, Conor R. Caffrey

**Affiliations:** 1 Center for Discovery and Innovation in Parasitic Diseases, University of California, San Francisco, San Francisco, California, United States of America; 2 Department of Pathology, University of California, San Francisco, San Francisco, California, United States of America; 3 Small Molecule Discovery Center, Department of Pharmaceutical Chemistry, University of California, San Francisco, San Francisco, California, United States of America; 4 Department of Pharmaceutical Chemistry, University of California, San Francisco, San Francisco, California, United States of America; 5 Department of Computer Science, San Francisco State University, San Francisco, California, United States of America; 6 Center of Infection and Immunity of Lille, Université Lille Nord de France, Inserm U1019, CNRS-UMR 8204, Institut Pasteur de Lille, Lille, France; 7 Department of Chemical Biology, GlaxoSmithKline, Research Triangle Park, North Carolina, United States of America; McGill University, CANADA

## Abstract

**Background:**

*Schistosoma* flatworm parasites cause schistosomiasis, a chronic and debilitating disease of poverty in developing countries. Praziquantel is employed for treatment and disease control. However, its efficacy spectrum is incomplete (less active or inactive against immature stages of the parasite) and there is a concern of drug resistance. Thus, there is a need to identify new drugs and drug targets.

**Methodology/Principal Findings:**

We show that RNA interference (RNAi) of the *Schistosoma mansoni* ortholog of human polo-like kinase (huPLK)1 elicits a deleterious phenotypic alteration in post-infective larvae (schistosomula or somules). Phenotypic screening and analysis of schistosomula and adult *S*. *mansoni* with small molecule inhibitors of huPLK1 identified a number of potent anti-schistosomals. Among these was a GlaxoSmithKline (GSK) benzimidazole thiophene inhibitor that has completed Phase I clinical trials for treatment of solid tumor malignancies. We then obtained GSKs Published Kinase Inhibitor Sets (PKIS) 1 and 2, and phenotypically screened an expanded series of 38 benzimidazole thiophene PLK1 inhibitors. Computational analysis of controls and PLK1 inhibitor-treated populations of somules demonstrated a distinctive phenotype distribution. Using principal component analysis (PCA), the phenotypes exhibited by these populations were mapped, visualized and analyzed through projection to a low-dimensional space. The phenotype distribution was found to have a distinct shape and topology, which could be elicited using cluster analysis. A structure-activity relationship (SAR) was identified for the benzimidazole thiophenes that held for both somules and adult parasites. The most potent inhibitors produced marked phenotypic alterations at 1–2 μM within 1 h. Among these were compounds previously characterized as potent inhibitors of huPLK1 in cell assays.

**Conclusions/Significance:**

The reverse genetic and chemical SAR data support a continued investigation of SmPLK1 as a possible drug target and/or the prosecution of the benzimidazole thiophene chemotype as a source of novel anti-schistosomals.

## Author Summary

Just one drug is available to treat schistosomiasis, a parasitic disease that affects hundreds of millions of people in developing countries. In the search for new drugs and drug targets, therefore, we have been interested in the schistosome version of human polo-like kinase (huPLK)1, an enzyme with critical functions in cell division. We used RNA interference to knock down messenger RNA for the SmPLK1 –the *Schistosoma mansoni* parasite’s version of huPLK1. This interference caused disruptive changes in the morphology of the immature ‘somule’ stage of the parasite, indicating that SmPLK1 is an important protein for survival. We then purchased, or acquired from GlaxoSmithKline (GSK), various small chemical inhibitors of huPLK1 and tested these against both the somules and adult parasites in culture. Many of these inhibitors caused severe changes in the parasite and, for somules, the differences could be computationally mapped and distinguished from unexposed parasites. For the GSK inhibitors, we observed ‘somule-adult bioactivity clustering,’ that is, chemicals active against the adults were also active against somules. This suggests that certain chemical attributes in the inhibitors are being favoured. Interestingly, many of the GSK inhibitors most active against the parasite are also known to both potently inhibit huPLK1 and kill cancer cells. Overall, our data suggest that SmPLK1 is a possible drug target and that the GSK chemistries could form the basis for developing a new drug to treat schistosomiasis.

## Introduction

Flatworm parasites of the *Schistosoma* genus are responsible for schistosomiasis, a chronic and often painful disease of poverty that affects more than 200 million people worldwide [1–3]. For over 35 years, treatment and control of this disease has relied on a single drug, praziquantel (PZQ) [4–6]. Apart from the concern over the possible emergence and establishment of resistance to this drug in the field [4, 7–9], PZQ has a number of other problems that encourage the search for alternate drugs. It is rarely curative at the single dose employed [10, 11] in part due to its rapid metabolism [12, 13], and the dose used is consequently high (40 mg/kg) relative to other oral anthelmintics and medications in general. Importantly, PZQ has diminished or no efficacy against developing schistosomes [14–16]. Finally, the drug has an unpalatable taste [17].

Efforts continue to identify and develop small synthetic compounds or natural products as anti-schistosomal drugs, *e*.*g*., [18–21]. In the hope of decreasing both the time and cost associated with developing drugs, researchers have also looked to either reposition registered drugs ‘as is,’ or use drugs or drug candidates as starting points for further chemical exploration and development [20, 22–25]. In this context, various anti-cancer small molecules, including those targeting components of the kinome [26] have been the subject of recent interest as potential anti-schistosomal drugs [24, 27–29]. Of these, a number of polo-like kinase (PLK) inhibitors, that either target the ATP-binding site [30–34] or the unique Polo-box domain [35, 36], have attracted our interest (see below) as a number of these are progressing pre-clinically or clinically as anti-cancer agents (S1 Table).

*S*. *mansoni* has just two PLK genes, *Smplk1* and *Smsak* (*Smplk4)* (GenBank IDs AAV49163 and GU084154, respectively), which is in contrast to the five found in humans [37–39]. PLKs are a family of conserved serine/threonine kinases, which, in humans, are involved in cell division, including G2/M transition, centrosome maturation, formation of bipolar spindles, cytokinesis and regulation of the spindle assembly checkpoint [40–43]. Plk1 is the best characterized member of the family and is vital to normal mitotic progression [40, 41, 44–46]. Its over-expression in human tumors [47–49] has identified this kinase as a selective target for anti-cancer drugs.

In *S*. *mansoni*, SmPLK1 is expressed in sporocysts (asexually dividing stages parasitizing the snail vector) and in adult worms, particularly in their reproductive organs, suggesting a contribution by this kinase to cell division [39]. The huPlk1 inhibitors, GW843682X and BI2536, are nanomolar inhibitors of SmPLK1 when the enzyme was expressed in *Xenopus* oocytes [39]. BI2536 also decreased the number of immature oocytes relative to mature oocytes in the female reproductive organs; in males, the size of testicular lobes and the number of spermatocytes were reduced [39]. Interestingly, SmSAK, which shares 37% and 13% identity in the kinase and polo-box domains, respectively, is not inhibited by BI2536 suggesting that the inhibitor is selective for SmPLK1 [39].

Using RNA interference (RNAi), we show that SmPLK1 and less so, SmSAK, are important to the normal development and survival of *S*. *mansoni* schistosomula (post-infective larvae, a.k.a. somules) in culture. Based on this finding, we then tested 11 clinically tracked inhibitors of huPLK1 for bioactivity on somules and adult parasites *in vitro*. One of these, a phase I clinical candidate benzimidazole thiophene inhibitor from GlaxoSmithKline (GSK), was bioactive at low micromolar concentrations. This inhibitor served as a starting point for a phenotypic screen of 38 benzimidazole thiophene analogs contained within GSKs Published Kinase Inhibitor Sets (PKIS) 1 and 2 [50–53]. We identify a structure-activity relationship (SAR) for this inhibitor set that is shared between somules and adult parasites, and we discuss our findings with a view to the possible future development of this compound class as a source of novel anti-schistosomals.

## Materials and Methods

### Ethics statement

Maintenance and handling of vertebrate animals were carried out in accordance with a protocol (AN107779) approved by the Institutional Animal Care and Use Committee (IACUC) at the University of California San Francisco. UCSF-IACUC derives its authority for these activities from the United States Public Health Service (PHS) Policy on Humane Care and Use of Laboratory Animals, and the Animal Welfare Act and Regulations (AWAR).

### Parasite material

A Puerto Rican isolate of *Schistosoma mansoni* was maintained by passage through albino *Biomphalaria glabrata* snails and infection of 3–5 week-old, female *Mesocricetus auratus* Golden Syrian hamsters [54, 55]. Cercariae (infectious larvae) were obtained from infected snails and mechanically transformed into somules as previously described [20, 56, 57]. To obtain adult schistosomes, hamsters were euthanized 42–45 days post-infection using an intra-peritoneal injection of 50 mg/kg sodium pentobarbital containing 50 U/ml heparin (as an anti-coagulant) in a total of 100 μL PBS. Worms were harvested by reverse perfusion of the hepatic portal system [54, 55, 58] in RPMI 1640 medium supplemented with 100 U/ml penicillin and 100 mg/ml streptomycin [54, 55]. Adults were transferred into Basch medium 169 [59] supplemented with 100 U/ml penicillin and 100 mg/ml streptomycin. In this medium, parasites were washed three times, allowed to stand for 30–60 min in the presence of 2X amphotericin B (fungizone) and then washed another three times in medium minus fungizone prior to phenotypic screening.

### Double-stranded (ds)RNA synthesis

Two dsRNA fragments, SmPLK1RNAi (358 bp) and SmPLK2RNAi (498 bp), that target the regulatory domain of the *Smplk1* gene transcript were generated by PCR using gene-specific primers containing a T7 promoter sequence (S2 Table) and the plasmid, SmPLK1-pcDNA3.1, as a template [39]. A similar strategy was employed for SmSAK-dsRNA, whereby two fragments of 722 bp and 473 bp were amplified from a SmSAK-pcDNA3.1 plasmid construct [37]. DsRNA synthesis employed the MEGAscript RNA Kit (Ambion) according to the manufacturer’s instructions. DsRNA was purified by precipitation with 3 M sodium acetate (pH 5.2) and ethanol, resuspended in dH2O, and quantified using a Nanodrop ND-1000 spectrophotometer (Nanodrop Technologies). The integrity of the dsRNA was confirmed by 1% agarose gel electrophoresis. DsRNA to the fluorescent *Discosoma sp*. mCherry protein was generated as a schistosome-unspecific control [20, 57].

### RNAi experiments

Co-incubation of somules with dsRNA was as described previously [20, 57]. Briefly, 300 somules were maintained at 37°C and 5% CO_2_ in 24-well plates (Corning Inc., 3544) containing 1 ml ‘complete’ Basch medium 169 supplemented with 100 U/ml penicillin, 100 mg/ml streptomycin and 5% FBS. DsRNA (30 μg/ml in 10–20 μl water) targeting SmPLK1, SmSAK or mCherry was added to the parasite cultures twice weekly out to 22 days. Cultures were observed daily for the appearance of phenotypes and experiments were performed twice each in duplicate.

To measure changes in gene expression as a consequence of RNAi, somules were co-incubated with the above dsRNA preparations for seven days. Experiments were performed twice each in duplicate. Parasites were then processed for reverse transcription-quantitative real time PCR (RT-qPCR) as described [20, 57].

### RNA isolation and gene expression analyses

These analyses were performed as described [20, 57, 60]. For RT-qPCR, total RNA was extracted using TRIzol reagent (Invitrogen) and the High Pure RNA Isolation Kit (Roche) following the manufacturer’s instructions. Complementary (c) first-strand DNA was synthesized using the SuperScript III First-Strand Synthesis kit (Invitrogen). cDNA was then used as a template for PCR amplification using the Light Cycler 480 SYBR green I Master mix (Roche) and a Mx3005P qPCR detection system (Stratagene). Specific primers for each of the regulatory and kinase domains of SmPLK1 and SmSAK, and for mCherry were designed using the Primer Express Program (Applied Biosystems; see S2 Table). Primers were experimentally validated and *S*. *mansoni* cytochrome C oxidase I (GenBank AF216698) was used as the reference transcript. Reactions were carried out in a final volume of 20 μl in 96-well plates (QPCR 96-Well Plates, Non-Skirted, Agilent Technologies). Experiments were performed twice each in duplicate. The 2^-ΔΔCt^ method [61] was employed to measure transcript levels post-RNAi and these were expressed as a percentage of those following exposure to mCherry dsRNA. Statistical analysis employed the two-tailed Student’s *t*-test. Quantification of mRNA from cathepsin B1.1 (AJ506157) was used as bystander control to monitor for off-target RNAi effects. Controls for genomic DNA contamination (no reverse transcriptase) and reagent purity (water control) were included for each sample.

### Phenotypic screening of somules and adults *in vitro* with small molecule inhibitors of huPLK1

Eleven inhibitors of huPLK1 were purchased. Of these, BI2536, BI6727, HMN-214 and MLN0905 were sourced from MedChemExpress; GSK461364, GW843682X, ON01910 and TAK960 were from AdooQBioScience LLC; thymoquinone was from Sigma, and poloxin and SBE13 were from Millipore. Inhibitors were dissolved in dimethyl sulfoxide (DMSO) at 10 or 20 mM stock concentrations which were stored at -20°C. A set of 38 benzimidazole thiophenes were also sourced as part of GSK’s Published Kinase Inhibitors Set (PKIS) 1 and 2 as 10 mM (10 μl) stocks in DMSO.

Phenotypic screens involving *S*. *mansoni* somules and adults were carried out as described [20, 21, 55, 62]. For somules, approximately 300 newly transformed parasites were dispensed into flat-bottomed 96-well plates in 100 μL complete Basch medium (Corning Inc., cat. # 3599). Compound was then added in a volume of 1 μl DMSO and the final volume brought up to 200 μL with complete Basch medium. Parasites were then incubated for up to two days at 37°C under 5% CO_2_. First pass, single concentration screens at 10 μM were performed and those compounds eliciting phenotypes were then re-screened over a concentration range of 0.5–10 μM (0.5% DMSO final).

For adult schistosomes, single concentration (10 μM) screens were performed in 24-well plates (Corning Inc., cat. # 3544) using five male worms per well in a final volume of 2 ml complete Basch medium. Compound was added in a volume of DMSO ranging from 0.5 to 2 μL. Those compounds eliciting phenotypes within 48 h were then re-screened over a concentration range of 1–10 μM.

### Automatic differentiation of the phenotypic response of somules

In the recent past, important advances have been made in algorithmic (automatic) phenotype analysis of somules; analogous methods do not yet exist for the adult stage of the parasite. In particular, methods have been developed for parasite segmentation [63–65], parasite tracking from video recordings [66] and quantitative identification of helminth phenotypes [63, 67], including for hit detection in high-throughput screens [68] and dose-response characterization [69]. As yet, however, no automated method exists for identification of phenotypes and simultaneous determination/scoring of their severity. Therefore, we combined automated phenotypic analysis with manual phenotype assessment in an integrated analysis process and applied it to analyze the phenotypes arising from exposure of the parasites to the commercially available inhibitors and the PKIS 1 and 2 inhibitors.

We began by automatically clustering the unaffected and affected juvenile parasites based on their differential phenotypic response. The existence of such a clustering and the fact that it could be identified without manual intervention, not only provided a rigorous and objective basis for the subsequent expert analysis, but also allowed us to quantify and visualize the phenotypic response space of the parasite.

For the above automated analysis, photographic images were taken at each time point and compound concentration using a Zeiss Axiovert 40 C inverted microscope and a Zeiss AxioCam MRc digital camera controlled by AxioVision 40 (version 4.8.1.0) software, as previously described [20, 55, 69]. The images were segmented (*i*.*e*., individual parasites were differentiated from background) using the Asarnow-Singh segmentation algorithm [64], to yield a total of 4,125 parasites (1,047 control and 3,078 drug treated) across the entire study. Subsequently, 11 descriptors were calculated for each segmented parasite. These descriptors included parasite area, length of the perimeter of the parasite, ratio of the major to the minor axis of the parasite body, ratio of the area of the parasite body to the area of its bounding box and a set of descriptors capturing the visual appearance of the parasite in terms of its intensity and texture. The parasite intensity was described using the mean and variance of the intensity distribution (we used the standard deviation as the specific numeric measure). Texture was described using Grey-Level Co-occurrence Matrices (GLCM), which capture how often two intensities occur side by side, and the following five descriptors (described further in Table 1): entropy, contrast, correlation, energy and homogeneity, which are computed on a normalized co-occurrence intensity matrix *I*(*i*, *j*). Further details on these descriptors as applied to parasitic screening can be found in [67].

**Table 1:** Phenotype descriptors to quantify parasite texture.

Entropy	$-\sum_{i=0}^{n-1}\sum_{j=0}^{n-1}I(i,j)\text{log}(I(i,j)$	Statistical measure of randomness related to the texture of an image.
Contrast	$\sum_{i=0}^{n-1}\sum_{j=0}^{n-1}\left(i-j)(i-j)I(i,j\right)$	The intensity contrast between a pixel and its neighbors in a region of the image.
Correlation	$\sum_{i=0}^{n-1}\sum_{j=0}^{n-1}\frac{\left(i-\mu_{i})(j-\mu_{j})I(i,j\right)}{\sigma_{i}\sigma_{j}}$	Measure of the linear dependency between the intensity values of pixels at particular positions relative to each other.
Energy	$\sum_{i=0}^{n-1}\sum_{j=0}^{n-1}I(i,j)I(i,j)$	The sum of the squared elements in the GLCM (gray-level co-occurrence matrix).
Homogeneity	$\sum_{i=0}^{n-1}\sum_{j=0}^{n-1}\frac{I(i,j)}{1+|i-j|}$	Measure of the closeness of the distribution of the elements in the GLCM to the GLCM diagonal.

### Defining and determining the severity of the phenotypic response by microscopical observation

The ‘traditional’ approach to adjudicating the many phenotypic responses possible for this parasite involves microscopical observation. We use simple ‘descriptors’ to record changes in movement, shape, translucence, surface integrity and, for adults specifically, the ability of the parasite to adhere to the culture dish surface (see S1 File and [20, 55, 70]). To convert these observations into an ordinal numeric output and thus facilitate relative comparisons of compound effects, each descriptor was awarded a ‘severity score’ of one up to a maximum score of four. When damage to the adult parasite’s tegument (surface) was evident, the maximum score of four was awarded on the assumption that such damage is lethal to the parasite, including in the mammalian host [14]. In the case of somules, phenotypes were recorded at 24 and 48 h; for adults, phenotypes were recorded at 1, 5, 24 and 48 h.

### Molecular modeling of the ATP-binding site of SmPLK1

To understand whether residues in the SmPLK1 ATP-binding site will accommodate the human benzimidazole thiophene PLK1 inhibitors, we built a homology model of SmPLK1 from the human ortholog’s structure (PDB ID: 2YAC). The homology model was constructed using the software PRIME (v.3.9; Schrödinger Inc). GSK benzimidazole thiophene inhibitors, GSK461364, GSK1030058, GSK326090 and GSK483724 were built using Maestro’s Edit/Build panel (v.10.1; Schrödinger Inc). LigPrep (v.3.3; Schrödinger Inc.) was used to minimize the ligand structures. We docked the ligands using Glide (v.6.6; Schrödinger Inc.) with the standard-precision docking scoring function. To compare the docking mode of the ligand GSK461364, we also docked it against the human PLK1 structure. Similarity between the docking poses was determined by evaluating the root-mean-square-distance (RMSD) of heavy atoms.

## Results

### RNAi of SmPlk1 and SmSAK induces abnormal phenotypes in S. mansoni somules

**Fig 1:**
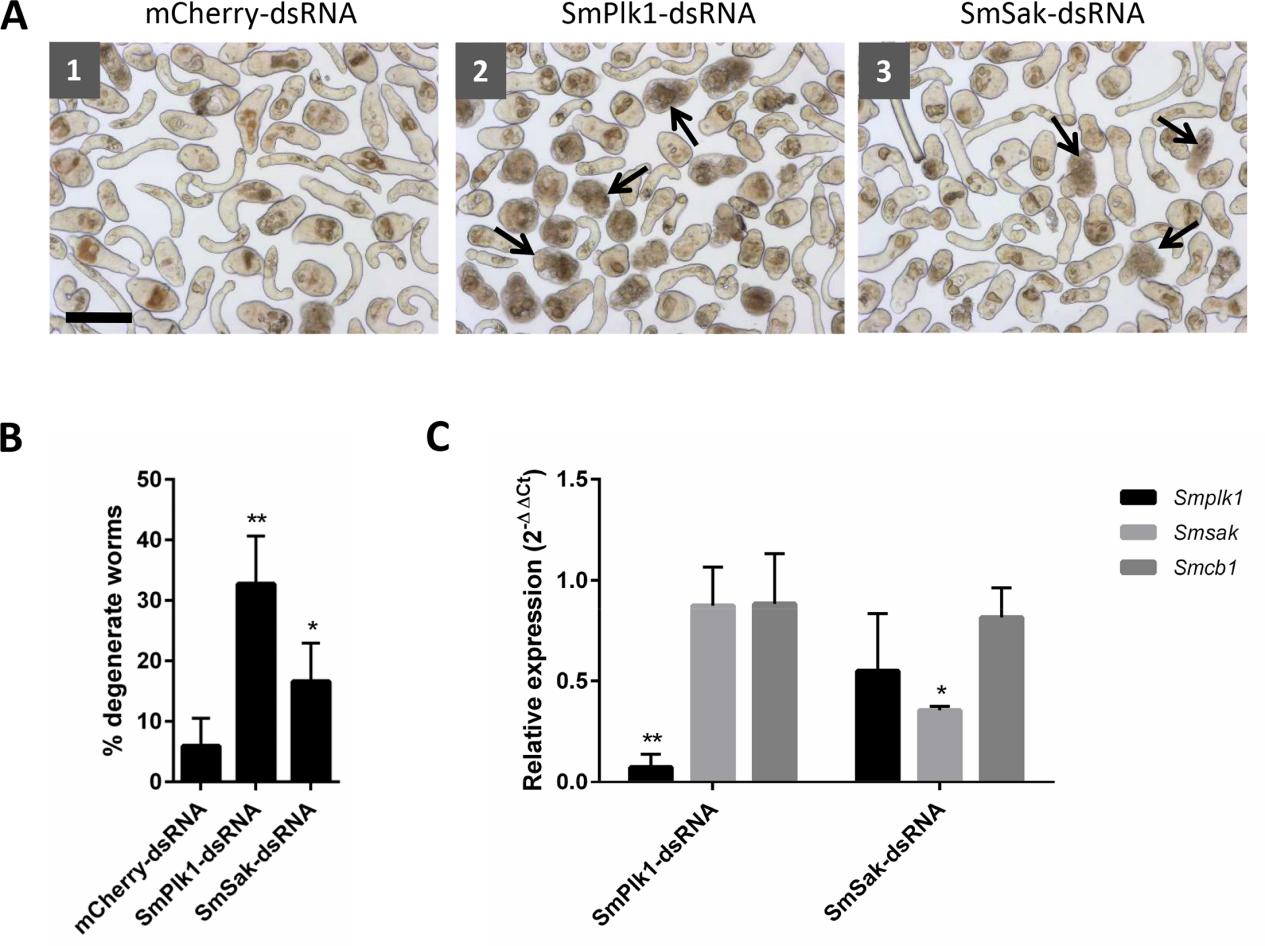
RNAi of SmPLK1 induces phenotypic changes in cultured *S*. *mansoni* somules. **A.** Somules were co-incubated for 22 days with dsRNA targeting (1) the non-schistosome mCherry protein as a control, (2) *Smplk1* or (3) *Smsak*. One representative image for each of the conditions is shown. Arrows point to degenerate parasites. Scale bar = 100 μm. **B.** Graph showing the percentage of degenerate worms. After 22 days of treatment, somules were counted by eye and those that were degenerate determined as a percentage of the total number of worms. Results are displayed as the mean +/- SD of two independent experiments each performed in duplicate (*p<0.05; **p ≤ 0.0005). **C.** Quantification of transcripts by RT-qPCR. Somules were co-incubated for seven days with dsRNA targeting transcripts of *smplk1 or smsak* and using dsRNA to mCherry protein as a non-schistosome control. For qPCR, *S*. *mansoni* cytochrome C oxidase I was used as a reference gene. *S*. *mansoni* cathepsin B1 *Smcb1* was used as a “bystander” gene to assess off-targeting by the dsRNA preparations of interest. Data are expressed using the 2^-ΔΔCt^ method as described in the Material and Methods: values represent the mean +/- SD of two independent experiments each performed in duplicate (*p<0.005; **p<0.001).

To evaluate whether SmPLK1 and SmSAK are important to the growth and/or survival of *S*. *mansoni* somules, we co-incubated the parasite with dsRNA targeting the respective gene transcripts. Parasites were exposed to 30 μg/ml dsRNA targeting the regulatory polo-box domain and the cultures observed every day for 22 days (Fig 1). In the presence of SmPLK1-dsRNA, rounding and darkening of the parasite were evident (Fig 1A, panel 2). Similar, but less pronounced, changes were also observed after co-incubation with SmSAK-dsRNA (Fig 1A, panel 3). After 22 days in culture, 32% and 16% of the somules exposed to SmPLK1- and SmSAK-specific RNAi, respectively, had been affected (Fig 1B). Quantification of RNAi was performed by RT-qPCR analysis of transcripts in parasites after seven days of incubation with the respective dsRNA preparations. These experiments indicated that expression of *Smplk1* and *Smsak* was decreased by 92.5 and 64.5%, respectively (Fig 1C). Expression of *Smcb1*, which we use as a ‘bystander’ gene to assess off-targeting by the dsRNA preparations of interest [20, 57] was not altered in the experiment. For adult parasites, we previously attempted RNAi of SmPLK1 via electroporation of 25 μg dsRNA but observed no phenotype after 5 days in culture [37].

### Differential phenotypic distribution of the control and treated parasite populations

We investigated the distribution of the phenotypes exhibited by the somules upon exposure to the 11 commercially available huPLK1 inhibitors and the 38 benzimidazole thiophene inhibitors available in PKIS 1 and 2. It may be noted that in phenotypic assays, it is common to observe differentiated responses of somules (and adult parasites) when exposed to drugs, even within a single well. This phenomenon can be due to natural variation of individuals, low drug concentration, or insufficient duration of exposure to the compound. The fact that schistosome clones do not exist, typically exacerbates phenotypic variability. The algorithmic analysis carried out in this paper highlights this issue through automated quantitative analysis.

**Fig 2:**
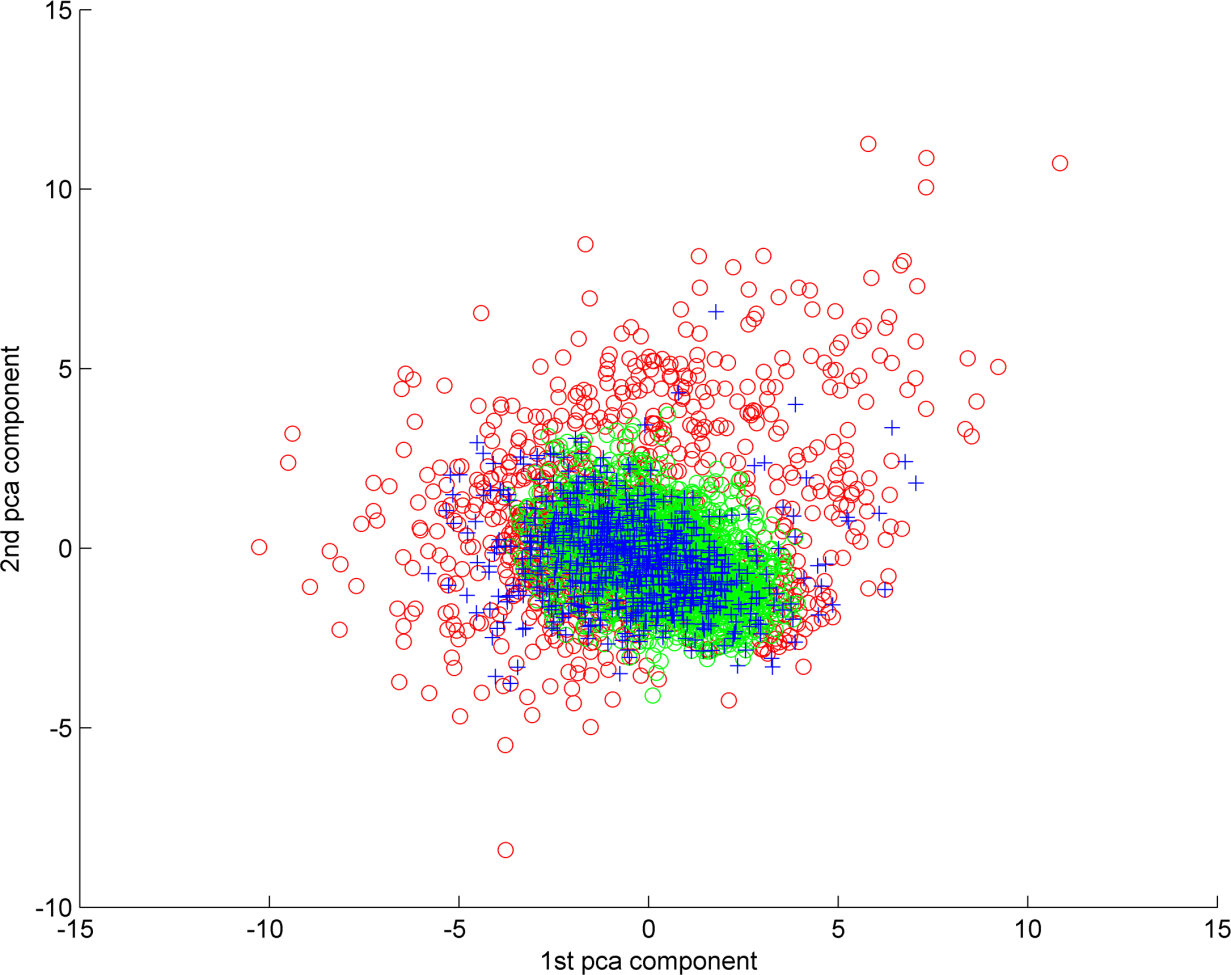
Differential distribution of parasites in terms of their shape- and appearance-based phenotypes as demonstrated by clustering. To enable visualization, the clustering results from the six-dimensional principal component analysis (PCA) space are mapped to a two-dimensional PCA space where the X-axis corresponds to the first PCA component and the Y-axis corresponds to the second PCA component. Control parasites are shown in blue. Parasites that were co-clustered with the controls but had been exposed to compounds are shown in green. These parasites did not exhibit significant phenotypic changes as a result of compound exposure and were consequently very similar to the control parasites. Parasites that had been exposed to compound and exhibited significant changes in their phenotypes are shown in red. A small number of control parasites (blue coloured “+” symbols) exhibited naturally occurring degeneracies and were placed outside the control cluster by the algorithm. Both the differential distribution and separability of the phenotypes exhibited by the control parasites from those exhibited by the treated parasites are obvious.

We employed cluster analysis to determine whether the unaffected and affected parasites could be automatically differentiated. For this purpose, based on the 11 computational descriptors of shape and appearance described earlier, each of the 4,125 parasites, identified after automatic segmentation, was represented as a point in an 11-dimensional phenotype (feature) space. To reduce dimensionality, we then projected the data to a lower dimensional feature space while retaining its variance, using principle component analysis (PCA). We mapped the data to a 6-dimensional PCA space, in which 95% of the variance in the data was accounted for. Next, we performed automatic clustering of the data using the *k*-means algorithm [71] (with the parameter k = 2, corresponding to the intuitive notion of separating the affected and unaffected parasites) to establish whether the phenotypes exhibited by the drug-treated parasites could be separated from those exhibited by controls. The clustering results produced two clear data groups (Fig 2). The first group formed a near compact core and corresponded to parasites from the control images (blue points) and also included parasites exposed to compounds that did not exhibit significant phenotypic changes, *i*.*e*., were similar to controls (green points). The second group was distributed around this core cluster and consisted of parasites that exhibited various phenotypic changes in their shape or appearance as a result of drug exposure (red points). Some of the parasites from the control images were also placed in this cluster due to naturally occurring degeneracies. Examples of individual parasites belonging to the different groups are presented in S1 Fig.

### Phenotypic screening of 11 commercially available Plk1 inhibitors

**Fig 3:**
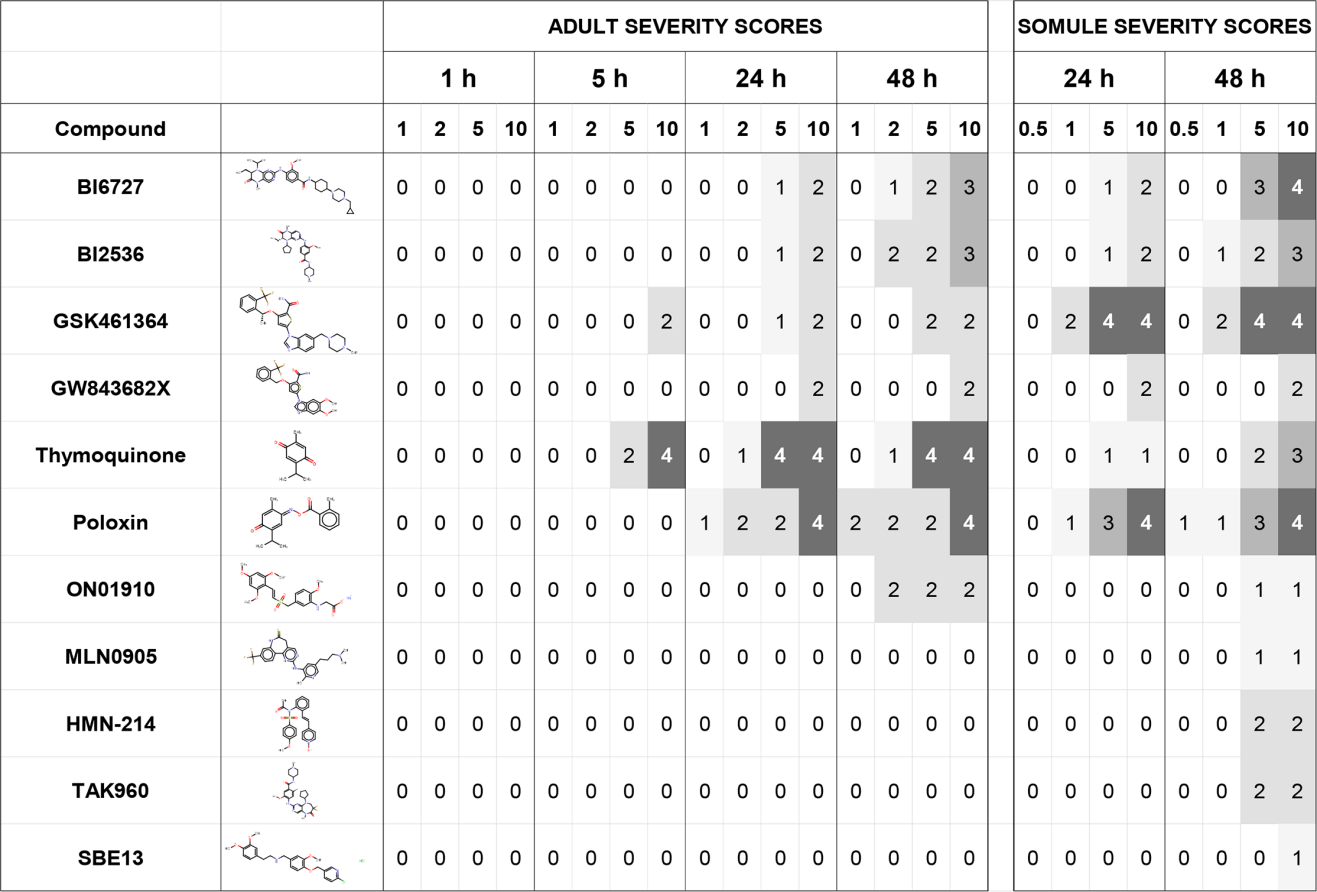
Phenotypic alterations in *S*. *mansoni* upon exposure to 11 commercially available human PLK1 inhibitors. Phenotypic alterations, expressed as severity scores (see Materials and Methods), were observed as a function of dose (1–10 μM) and time (up to 48 h). Severity scores ranged from 0 (no effect) to a maximum of 4. S1 File (worksheets 1 and 2) lists the descriptors that determine the severity scores calculated (worksheet 3).

All of the inhibitors eliciting phenotypic changes in the parasite as determined visually did so in a concentration- and time-dependent manner (Fig 3 for severity scores and S1 File, worksheets 1–3 for both the descriptors and severity scores). The ATP-competitive dihydropteridinone inhibitor, BI2536, and its successor BI6727, which displays improved pharmacokinetic, efficacy and safety profiles [72–74], were similarly active against adults and somules whereby phenotypic alterations were first noted at 5 μM after 24 h. The ATP-competitive benzimidazole thiophenes, GSK461364 and GW843682X, diverged in their relative potencies. The former was much more active against both developmental stages, particularly the somules, for which the inhibitor was the most potent of 11 inhibitors tested (multiple phenotypic changes noted at 1 μM after 24 h). This is perhaps not surprising, as GW843682X was an early tool molecule with modest cellular activity, whereas GSK461364 was a clinical candidate [51–53, 75, 76]. The natural product, thymoquinone, and its synthetically derived analog, poloxin, both of which target the unique polo-box domain of huPLK1 [77], were also potent anti-schistosomals: thymoquinone exerted preferential activity against adults (a severity score of 2 at 5 μM after 5 h). The other five inhibitors (ON01910, MLN0905, HMN-214, TAK960 and SBE13) displayed little to no activity (severity scores of 1–2 only after 48 h).

### Phenotypic screening of 38 benzimidazole thiophene inhibitors

**Fig 4:**
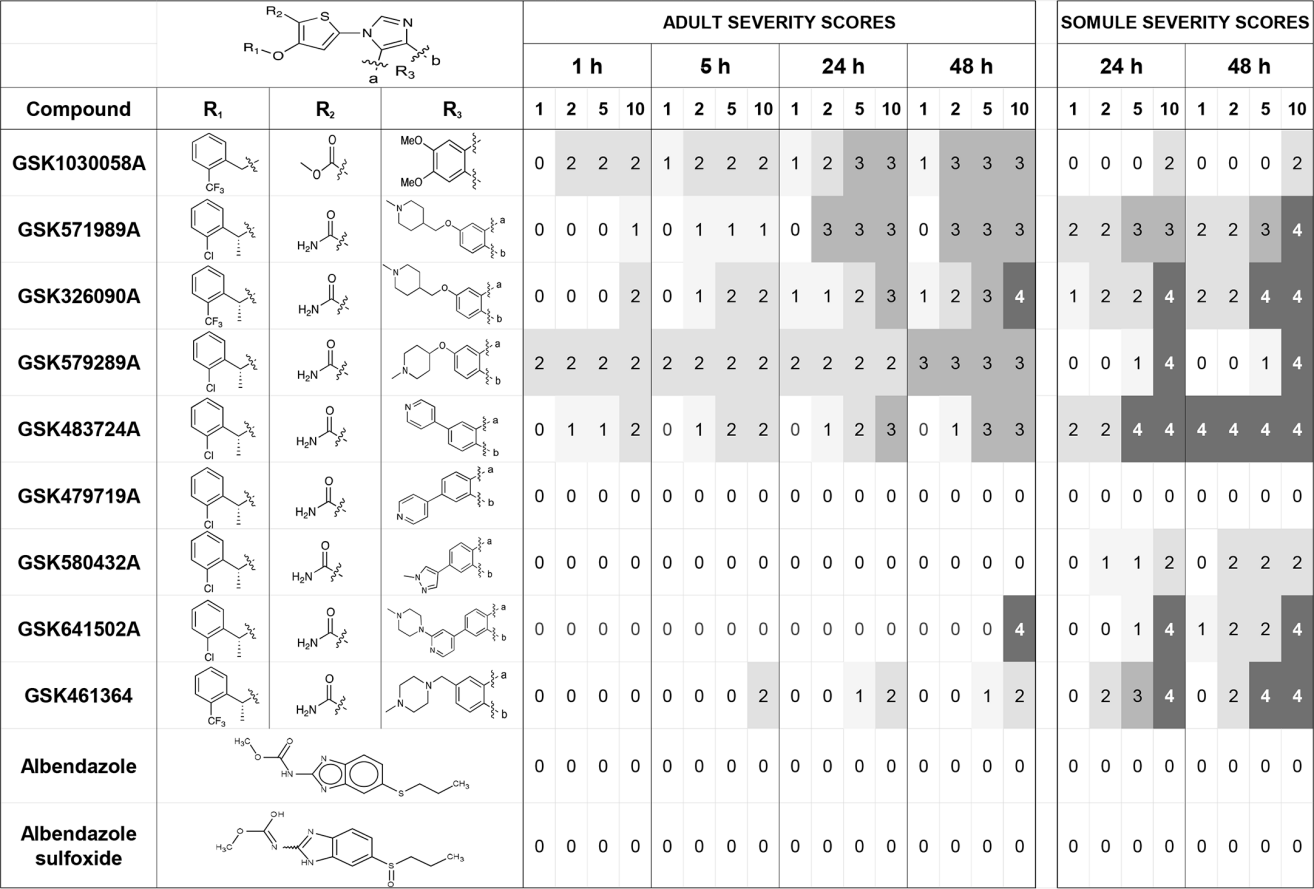
Phenotypic alterations in *S*. *mansoni* upon exposure to GSK’s benzimidazole thiophene PLK1 inhibitors. Phenotypic alterations, expressed as severity scores (see Materials and Methods), were observed as a function of dose (1–10 μM) and time (up to 48 h). Severity scores ranged from 0 (no effect) to a maximum of 4. A selection of data for those most potent inhibitors is presented. The full dataset, including descriptors for the 38 compounds tested, is presented in S1 File, worksheets 4–6).

For both somules and adults, Fig 4 depicts a snapshot of the severity scores as a function of time and concentration for the 38 benzimidazole thiophenes found within GSKs PKIS 1 and 2. The complete data set (descriptors and severity scores) is provided in S1 File (worksheets 4–6). For the bioactive compounds, the most common phenotypic response noted for adult parasites involved overactive uncoordinated movements whereby the parasites displayed rhythmic movements being unable to adhere to the bottom of the culture well. For some compounds, *e*.*g*., GSK1030058A and GSK579289A, this response appeared rapidly (within 1 h) at 1 or 2 μM. The uncoordinated response generally progressed during the two day incubation period to include a loss of translucency (darkening) sometimes accompanied by worm shrinkage, each of which increased the overall severity score. For the 21 benzimidazole thiophenes present only in the PKIS 1 and screened previously against adults [29] under assay conditions described earlier [78], 14 bioactive compounds were shared (see S1 File worksheets 4 and 6).

For somules, concentration- and time-dependent changes in the parasites were also evident of which over-activity and a general darkening and rounding of the parasite were the most common. For the most potent compounds, multiple phenotypic changes were noted at 1 μM by the first time-point of 24 h. For some compounds at 48 h, internal disruption was evident by the appearance of multiple ‘vacuoles’ (e.g., GSK483724A; and GSK641502A; S2 Fig).

Also evident from Fig 4 and S1 File is a clustering of active and inactive benzimidazole thiophenes for both adults and somules (SAR detailed below) with some exceptions. This clustering may indicate a shared target(s) and/or mechanism(s) of action between both developmental stages which would be encouraging from the point of view of developing a compound that possesses bioactivity across the entire developmental cycle of the parasite in the mammalian host.

### Detailed SAR

S1 File should be used to adjudicate the SAR. The predominant substituents at R1, R2 and R3 for the 38 available benzimidazole thiophenes were a 2-trifluoromethylbenzyl or 2-chlorobenzyl (21/38 compounds), a primary amide (33/38), and a 5,6-dimethoxybenzimidazole (18/38), respectively. Of 58 thiophene benzimidazoles synthesized and reported in [51], these particular R1-R3 substitutions yielded the lowest IC_50_ values against both the target huPLK1 enzyme (2 nM) and the HCT116 human colon carcinoma cell line (699 nM) used to assess cellular activity.

Focusing first on the adult responses, the cut-off we assigned to indicate compound activity was the annotation of one or more phenotypic changes at 10 μM by 24 h. With the above stated R1 and R3 sub-structures fixed, R2 as a methyl ester (compound ID ending in 0058A), methyl amide (0061A), dimethylamide (0062A) or primary amide (3682X and 2849X) maintained activity against the parasite whereas the methyl ketone (0059A) was inactive. Of the R2 substitutions tested, the methyl ester was the most potent with activity recorded at 2 μM after 1 h compared to 5 or 10 μM after 24 h for the others. This is interesting in that GSK reported the methyl ester to be essentially inactive against the huPLK1 (IC_50_ > 1 mM) [51]. The potent activity against the parasite could be explained if, under these assay conditions, the ester is hydrolyzed to the carboxylic acid which does inhibit PLK1, or if the ester is an inhibitor of an important, but as yet unknown, target. Apart from 0058A, SAR in this area proved to be insensitive to the presence or absence of hydrogen bond donors, however the loss of the polar terminus, in the case of 0059A, showed that the presence of a hydrogen bond acceptor alone is not sufficient to retain activity.

Continuing with R2 fixed as the primary amide, the presence of a methyl group at the benzylic position of the terminal R1 ring system 7701A(*R*) and 7700A(*S*) improved bioactivity compared to 2849X which does not have a methyl group in the benzylic position. The original GSK report highlights this change as one that can enhance cellular activity [52]. Compounds such as 7314A and 7315A with mono-methoxy benzimidazoles retained activity against the parasite. In the context of the mono-methoxy benzimidazoles, both an ortho CF3 group (7315A) and an ortho Cl group at R1 were active.

Focusing on R1, replacing the 2-trifluoromethylbenzyl or 2-chlorobenzyl with a 3-choro-(thiophen-2-ylmethoxy) (8459A) retains activity at least transiently, but in the absence of the halogen, the 2-thiophen-2-ylmethoxy alone (2948A) is inactive. Also inactive at R1 are a 2-furylmethoxy (4607A), cyclohexylmethoxy (4559A) and a 4-pyridinylmethoxy (6313A) even when the latter has an additional bromine at position 2 (4278X). Compounds with an additional one (4925A) or two methylene linkages (5189A) extending to the terminal R1 phenyl group are not active. Likewise, the R1 phenyl containing a 4-methyl sulfone (9979X) or the same group at position 2 in the presence of an R2 cyano group (9347A) is inactive. Overall, the data thus far indicate the importance of an electronegative group at R1. Perhaps there is a columbic attraction at play in a binding pocket making this advantageous for binding.

Continuing on with a 2-trifluoromethylbenzyl or 2-chlorobenzyl and the primary amide fixed at R1 and R2, respectively, the effects of altering (extending) R3 were pronounced and indeed yielded the most active compounds tested. For huPLK1, these substituents likely extend towards solvent, and can be used to modulate solubility and other chemical properties, in addition to potency for the enzyme. Thus, with a 4-(1-methylpiperidin-4-yl)methyl at R3, the 2-trifluoromethylbenzyl at R1 (6090A) was as potent as the 2-chlorobenzyl (1989A), *i*.*e*., bioactivity was discernible at 10 μM after just 1 h. Shortening the R3 to a 4-(1-methylpiperidin-4-yl) moiety (9289A) produced the most potent compound tested whereby activity was recorded at 1 μM after 1 h. Strong potency was retained by substituting the 4-pyridin-4-yl group but only when it originated from the 6 position (3724A). Placing the same 4-pyridin-4-yl group in the 5 position (9719A) resulted in the complete loss of activity against the parasite. Compounds 0432A, 1502A, and 7232A also have substituents appended in the 5-position and they too show little or no activity against the parasite. The current clinical candidate GSK461364 containing a terminal 4-methyl piperazine was active with bioactivity detectable at 5 μM after 24 h. Not all polar substituents in the 6 position retained activity, however. The diol 8744A, for example, was inactive against the parasite. Finally, compound 6294A with a lipophilic t-butyl urea substituent in the 6 position was active.

The last groups of compounds include relatively simple versions of this chemotype, with no groups in the 5 and 6 positions of the benzimidazole. They are generally inactive against the parasite. For example, when R1 is a 2-fluorobenzyl, 2-bromobenzyl or 4-methoxyphenyl group (4306A, 3156A and 4304A) and there is no substitution of R3, the compounds are inactive. In contrast, a 3-methoxyphenyl variant (4482X) at R1 was active, albeit only at 10 μM. With a 2-bromophenyl (3609X) or 2-methoxyphenyl group (3349X) at R1, R3 substitution at the 5 position with trifluoromethyl or chloro substituents, respectively, resulted in no activity, whereas the 6-trifluoromethyl variant (3606X) possessed some activity.

With some exceptions, *e*.*g*., 2948A and 4925A, the compounds active against the adults were also active against the somules (using the same cut-off for activity, *i*.*e*., 10 μM by 24 h). Notable was the fact that the addition of the methyl group proximal to the terminal R1 group enhanced activity against somules but did not greatly influence activity against adults (compare 2849X with both 7701A(*R*) and 7700A(*S*)). Lastly, neither the antinematodal benzimidazole drug, albendazole, nor its sulfoxide metabolite, was active over the concentrations tested.

Based on the above analysis of 38 benzimidazole thiophenes in the PKIS 1 and 2 libraries, an optimized structure emerges for further SAR: a 2-trifluoromethylbenzyl or 2-chlorobenzyl at R1, a primary carboxamide or methyl ester at R2 and bi-aryl rings at R3 decorated with solubilizing aliphatic amines. Continued exploration of the influence of halogen substitutions on different positions of the R1 ring would be warranted to understand whether efficacy can be improved. Also, for R2, methyl esters present pharmacokinetic (PK) liabilities and are prone to hydrolysis in aqueous media, and, thus, would need to be avoided in favor of the primary amide common to most of the compounds tested here. If, indeed, the methyl ester serves as a pro-drug for an active carboxylic acid, this could be explored in more detail to optimize the release of the acid. For R3, the bi-aryl R3 substitutions yielded a cluster of potent compounds, including two (9289A and 3724A) that were active at 2 μM or less after 1 h, and that induced progressively more severe phenotypic disturbances.

### Similar binding pose of a benzimidazole thiophene in the ATP-binding sites of human and schistosome PLK1

**Fig 5:**
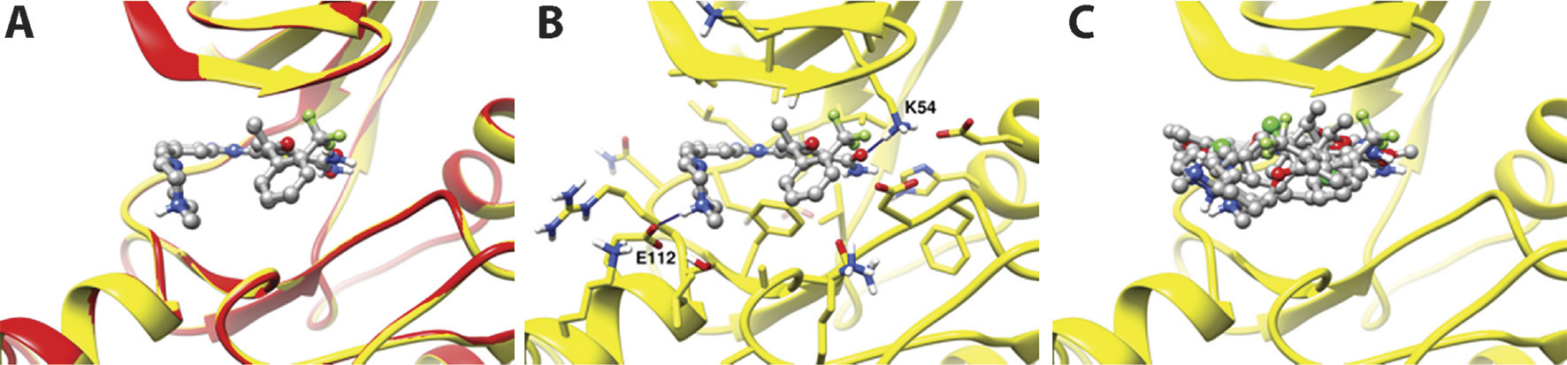
Binding modes of GSK461364 in human and *S*. *mansoni* PLK1. **A.** Docking pose of GSK461364 in the huPLK1 crystal structure (red color) and SmPLK1 model (yellow color). GSK461364 is shown in ball-and-stick representation. **B**. Docking pose of GSK461364 in the SmPLK1 binding pocket. GSK461364 makes hydrogen bonding interactions with Lys54 and Glu112 residues (blue colour lines). Carbon, nitrogen, oxygen and hydrogen atoms of SmPLK1 are shown in yellow, blue, red and white colours, respectively. GSK461364 is shown in ball-and-stick representation. **C**. Docking poses of GSK inhibitors GSK461364, GSK1030058, GSK326090, and GSK483724 in the SmPLK1 model. GSK inhibitors are shown in ball-and-stick representation. Carbon, nitrogen, oxygen, sulphur and hydrogen atoms of GSK inhibitors are shown in gray, blue, red, yellow and white colours, respectively.

In order to assess the binding mode of the benzimidazole thiophenes, we docked a representative inhibitor, the clinical Phase I drug candidate, GSK461364, in the ATP-binding site of the huPLK1 structure (pdb id: 2yac) and in an homology model of SmPLK1. The predicted binding poses, shown in Fig 5A, are very similar (0.7Å heavy atom RMSD), as are the Glide-SP docking scores, which were -9.3 and -9.6 for the human and *S*. *mansoni* enzymes, respectively. Thus, the data from the molecular modeling calculations suggest that the current benzimidazole thiophenes bind to the SmPLK1 without undue energy penalties, as observed by their similar docking scores and predict that the binding orientation will be very similar between huPLK1 and SmPLK1. A close-up view of the GSK461364 binding to SmPLK1 (Fig 5B) shows that the inhibitor makes hydrogen bonding interactions with Lys54 and Glu112 residues. Similar interactions were also observed for GSK461364 docked to huPLK1 (not shown). Further, the docking poses of GSK461364 and three other GSK benzimidazole thiophene inhibitors (GSK483724, GSK1030058 and GSK326090) against SmPLK1 show that the benzimidazole thiophenes bind in the same orientation for all the inhibitors (Fig 5C).

## Discussion

Reliance on a single drug to treat ‘continents’ of people afflicted with schistosomiasis encourages the search for new drugs and drug targets [6, 11, 79]. RNAi has proven to be a key research tool in this endeavor by identifying gene products that are essential to parasite survival (*e*.*g*., [20, 29, 57, 80, 81]). In this report, we demonstrate that RNAi of the single gene schistosome ortholog of huPLK1 leads to degenerative changes in the morphology of *S*. *mansoni* somules. Our findings are consistent with those very recently reported by Bickle and colleagues [29] as are our previous unsuccessful attempts to discern phenotypes consequent on RNAi of SmPLK1 in adult schistosomes [39]. Because somules undergo major transformative changes as they adapt to the mammalian host, it’s possible that they are more sensitive to perturbations in gene expression than adults. With the knowledge that SmPLK1 contributes to survival and that huPLK1 is a well-validated drug target for treatment of various cancers [30, 32, 48], we obtained pre-clinically and clinically advanced small molecule inhibitors of huPLK1 that might form starting points for the development of novel anti-schistosomals.

The algorithmic analysis of somule phenotypes upon exposure to the commercially available and PKIS benzimidazole thiophenes produced two significant results. First, it demonstrated an objective distinction between phenotypes of affected and unaffected somules, and that drug exposure leads to distinct phenotypic effects, which were identified and quantified without recourse to (subjective) human intervention and perceptual analysis. Second, we were able to quantify and visualize the phenotype space of the parasite through low-dimensional projections. In the low-dimensional space, the distribution of the phenotypes was not random, but was found to have a distinct shape and topology. Our analysis focused on somules owing to the lack of automated techniques for segmenting and phenotyping adult parasites. Part of our ongoing research involves the development of algorithms to enable a similar analysis of the phenotypic response space of adults.

We first tested 11 commercially available PLK1 inhibitors, including a number that are in clinical trials, for their activity against both somules and adults. A range of phenotypic responses, from pronounced deleterious changes at low micromolar concentrations to complete inactivity, were recorded. The benzimidazole thiophene, GSK461364, was the most potent of the inhibitors tested against somules and generated early (within 5 h) and sustained alterations in the adult parasite. GSK461364 is a single digit nanomolar inhibitor of huPLK1 and other PLK isoforms, and is at least 100-fold less potent against non-PLK kinases [52, 75, 76, 82]. The drug candidate is also a low micromolar inhibitor of cancer cell line proliferation from multiple origins with minimal toxicity to non-dividing human cells [82]. GSK461364 has successfully completed Phase I clinical trials for treatment of specific advanced solid tumors and Non-Hodgkin’s Lymphoma [83]. The clinical progress of GSK461364 along with the recent availability of 38 structurally related benzimidazole thiophenes within GSK’s PKIS 1 and 2 libraries [50–53] prompted us to explore: (i) whether more effective inhibitors than GSK461364 against the parasite exist, (ii) whether an SAR for anti-parasitic activity could be identified, and (iii) whether any SAR was similar to that demonstrated for huPLK1 by previous GSK research [51–53]. Satisfying the first two conditions would be of interest in having introduced and characterized a new anti-schistosomal chemotype yet, in the absence of particular knowledge of the molecular target or mechanism of action, would create a more challenging, but not insurmountable (e.g., [84–87]), situation for chemical optimization. Meeting all three conditions, however, would support a decision to initiate a target-based SAR program centered on SmPLK1 which would include recombinant expression, purification and crystallography of SmPLK1 in order to drive the iterative chemical optimization process. Such a program would be aided by the considerable metabolism and toxicity data that are available for many of the benzimidazole thiophenes [52, 53].

A number of the 38 benzimidazole thiophenes tested induced phenotypic changes in the parasite that increased in severity as a function of time and concentration. Compound clusters with bioactivity against both adults and somules were identified. For adults, the main initial response recorded for this chemotype was uncoordinated over-activity with an inability of the parasite to adhere to the dish, perhaps suggesting a disruption in neuromuscular homeostasis. A cluster of four R3 bi-aryl compounds (GSK571989A, GSK326090A, GSK579289A and GSK483724A) was especially potent, inducing uncoordinated over-activity at just 1 or 2 μM within 5 h for adults. For somules, these same compounds induced multiple and progressively more severe responses in the parasite (*e*.*g*., over-activity and internal degeneracy). The data are encouraging given the need to identify anti-schistosomal compounds that target across the spectrum of developmental stages of the parasite in the human host, which is a key failing of the current drug, PZQ [14]. These bioactive compounds are not significantly dissimilar in structure from the clinical candidate GSK461364 and are part of a sub-series of compounds designed to explore solubility and/or limit CYP-mediated metabolism [53]. For example, GSK483724A (compound 14 in [53]) displays IC_50_ inhibition values of 0.1 μM or less for the major drug-metabolizing CYP450 isoforms, CYP2C9 and CYP3A4. Thus, further exploration of an expanded R3 bi-aryl substituent series, including those presented in [53] would be worthwhile.

Whether or not these early uncoordinated responses in the adult parasite prove relevant for *in vivo* efficacy remains to be seen, however, it is pertinent to note that, for helminths, interference with neuromuscular activity (if indeed that is occurring here) is a well-proven anthelmintic strategy [88]. Overall, it is noteworthy that the progressive phenotypic disturbances recorded for both adults and somules occur at low micromolar, and potentially sustainable, plasma concentrations of compounds.

Some correlation exists between our data for bioactivity of the PKIS 1 and 2 benzimidazole thiophenes against the parasite and those published for inhibition of the huPLK1 enzyme and growth of a human HCT116 colon carcinoma cell line [51–53] (summarized in S1 File, worksheet 6). In spite of the gaps in the published data, we note that those PLK1 inhibitors generating the lowest nanomolar IC_50_ values against both huPLK1 and the HCT116 cells were also those most active against the parasite irrespective of the developmental stage tested (*e*.*g*., compare 6090A and 9289A vs. 6313A and 3606X). Although not proof that SmPLK1 is the relevant target of these inhibitors, the data are suggestive. Also, from a molecular perspective, the DOCKing results with representative benzimidazole thiophenes suggest that SmPLK1 should be potently inhibited.

The above observations support an attempt to heterologously express SmPLK1 in order to understand whether the relationship noted at the whole organism level can be substantiated at the level of the respective PLK enzymes. HuPLK1 has been successfully expressed in baculovirus-infected *Trichoplusia ni* cells [82]. We do not discount the possibility that the activities noted against the parasite are due to off-targeting, *i*.*e*., compound interactions in addition to or apart from inhibition of SmPLK1. Indeed, it is difficult at this juncture to reconcile the rapid onset of uncoordinated motility upon exposure to some of the benzimidazole thiophenes with the intended molecular target that has a highly constrained activity during mitosis. Again, recombinant expression of the target schistosome enzyme would be important to address whether the whole organismal effects noted here correlate with kinase inhibition.

To conclude, SmPLK1 is an essential gene for the somule stage of *S*. *mansoni*. Based on the druggability of the human ortholog in anti-cancer chemotherapy, we phenotypically screened commercially available PLK1 inhibitors and a series of 38 benzimidazole thiophenes present in GSK’s PKIS 1 and 2. An SAR across somules and adults was observed for the benzimidazole thiophenes, particularly for substitutions off the bi-aryl system at R3 which yielded fast-acting and potent compounds that merit further exploration. The apparent correlation between the present anti-parasite data and those noted previously for inhibition of PLK1 in human cancer cells suggests that SAR studies with the respective human and schistosome PLK orthologs should be considered.

## Supporting Information

S1 Table Small molecule inhibitors of human PLK1 in development. (DOCX)

S2 Table Primers for dsRNA and qPCR. (DOCX)

S1 File Screening and structure bioactivity relationship for 38 PKIS benzimidazole thiophenes. (XLSX)

S1 Fig Differential phenotypic distribution of control and treated parasite populations. Examples of control parasites are shown in panels 1A through C. These parasites were also algorithmically grouped in a cluster that showed little or no phenotypic changes (blue in Fig 2). Panels 2A-C: examples of parasites deemed by the clustering algorithm to be unaffected by compound action (depicted in green in Fig 2). Of these, the parasites shown in Panels 2A, B and C were exposed to GSK448459A, GSK1030059A and GSK580432A, respectively. Note that even though these compounds were weakly active in terms of their effects on the parasite population, not every parasite was equally or significantly affected by the compound. Such phenotypic heterogeneity is commonly encountered as demonstrated here. Panels 3A-C: examples of parasites affected by compounds as determined both by algorithm clustering and manual scoring (represented by red points in Fig 2). The parasites shown in Panels 3A, B and C were exposed to GSK483724A, GSK641502A and GSK346294A, respectively. These compounds were also found to be active by manual scoring.
(TIF)

S2 Fig Example phenotypic effects of PKIS 1 and 2 benzimidazole thiophenes on somules. Parasites were incubated for 48 h in the presence of the appropriate DMSO control (A) or 1 μM GSK483724A (B) or GSK641502A (C). Note the internal vacuolization in parasites exposed to GSK compounds. Bar = 200 μM.
(TIF)
