# Supplementary material for: Structure-Bioactivity Relationship for Benzimidazole Thiophene Inhibitors of Polo-Like Kinase 1 (PLK1), a Potential Drug Target in Schistosoma mansoni
Source: PLoS Negl Trop Dis. 2016 Jan 11;10(1):e0004356. doi: 10.1371/journal.pntd.0004356 (PMC4709140; doi:10.1371/journal.pntd.0004356)
Supplement: S1 Table — (DOCX) [file pntd.0004356.s001.docx]

| **Inhibitor** | **Clinical phase** | | | | **References** |
| --- | --- | --- | --- | --- | --- |
|  | Pre-clinical | Phase I | Phase II | Phase III |  |
| **BI6727**  **(Volasertib)** |  |  |  |  | [1, 2, 3] |
| **BI2536** |  |  |  |  | [4] |
| **GSK461364** |  |  |  |  | [4, 5] |
| **GW843682X** |  |  |  |  | [6] |
| **Thymoquinone** |  |  |  |  | [7] |
| **Poloxin** |  |  |  |  | [7] |
| **ON01910**  **(Rigosertib)** |  |  |  |  | [8, 9, 10] |
| **MLN0905** |  |  |  |  | [10, 11] |
| **HMN-214** |  |  |  |  | [10, 11] |
| **TAK-960** |  |  |  |  | [10, 11] |
| **SBE13** |  |  |  |  | [10, 11] |

1. O'Neil BH, Scott AJ, Ma WW, Cohen SJ, Aisner DL, Menter AR, et al. A phase II/III randomized study to compare the efficacy and safety of rigosertib plus gemcitabine versus gemcitabine alone in patients with previously untreated metastatic pancreatic cancer. Ann Oncol. 2015. pii: mdv264. PubMed PMID:26091808.
2. Gjertsen BT, Schoffski P. Discovery and development of the Polo-like kinase inhibitor volasertib in cancer therapy. Leukemia. 2015;29(1):11-9. doi: 10.1038/leu.2014.222. PubMed PMID: 25027517; PubMed Central PMCID: PMC4335352.
3. Rudolph D, Impagnatiello MA, Blaukopf C, Sommer C, Gerlich DW, et al. Efficacy and mechanism of action of volasertib, a potent and selective inhibitor of Polo-like kinases, in preclinical models of acute myeloid leukemia. J Pharmacol Exp Ther. 2015;352(3):579-89. doi: 10.1124/jpet.114.221150. PubMed PMID: 25576074.
4. Strebhardt K, Becker S, Matthess Y. Thoughts on the current assessment of Polo-like kinase inhibitor drug discovery. Expert Opin Drug Discov. 2015;10(1):1-8. doi: 10.1517/17460441.2015.962510. PubMed PMID: 25263688.
5. Olmos D, Barker D, Sharma R, Brunetto AT, Yap TA, Taegtmeyer AB, et al. Phase I study of GSK461364, a specific and competitive Polo-like kinase 1 inhibitor, in patients with advanced solid malignancies. Clin Cancer Res. 2011;17(10):3420-30. doi: 10.1158/1078-0432.CCR-10-2946. PubMed PMID: 21459796.
6. Lansing TJ, McConnell RT, Duckett DR, Spehar GM, Knick VB, Hassler DF, et al. In vitro biological activity of a novel small-molecule inhibitor of polo-like kinase 1. Mol Cancer Ther. 2007;6(2):450-9. doi: 10.1158/1535-7163.MCT-06-0543. PubMed PMID: 17267659.
7. Reindl W, Yuan J, Kramer A, Strebhardt K, Berg T. Inhibition of polo-like kinase 1 by blocking polo-box domain-dependent protein-protein interactions. Chem Biol. 2008;15(5):459-66. doi: 10.1016/j.chembiol.2008.03.013. PubMed PMID: 18482698.
8. Ellis PM, Leighl NB, Hirsh V, Reaume MN, Blais N, Wierzbicki R, et al. A Randomized, Open-Label Phase II Trial of Volasertib as Monotherapy and in Combination With Standard-Dose Pemetrexed Compared With Pemetrexed Monotherapy in Second-Line Treatment for Non-Small-Cell Lung Cancer. Clin Lung Cancer. 2015. pii: S1525-7304(15)00142-4. doi: 10.1016/j.cllc.2015.05.010. PubMed PMID: 26100229.
9. Chun AW, Cosenza SC, Taft DR, Maniar M. Preclinical pharmacokinetics and in vitro activity of ON 01910.Na, a novel anti-cancer agent. Cancer Chemother Pharmacol. 2009;65(1):177-86. doi: 10.1007/s00280-009-1022-9. PubMed PMID: 19466411
10. Garuti L, Roberti M, Bottegoni G. Polo-like kinases inhibitors. Curr Med Chem. 2012;19(23):3937-48. PubMed PMID: 22709006.
11. Schöffski P. Polo-like kinase (PLK) inhibitors in preclinical and early clinical development in oncology. Oncologist. 2009;14(6):559-70. doi: 10.1634/theoncologist.2009-0010. PubMed PMID: 19474163.
