# Supplementary material for: Structure-Bioactivity Relationship for Benzimidazole Thiophene Inhibitors of Polo-Like Kinase 1 (PLK1), a Potential Drug Target in Schistosoma mansoni
Source: PLoS Negl Trop Dis. 2016 Jan 11;10(1):e0004356. doi: 10.1371/journal.pntd.0004356 (PMC4709140; doi:10.1371/journal.pntd.0004356)
Supplement: S2 Table — (DOCX) [file pntd.0004356.s002.docx]

| **Name** | **Target** | **Sequence (nucleotides (nt) covered)** |
| --- | --- | --- |
| **Primers for dsRNA synthesis ^a^** | | |
| SmPLK1T7F | SmPLK1 | 5’-TAATACGACTCACTATAGGGGTGGTGCCCTTGGTAATGTCGG-3’ (nt 971-992) |
| SmPLK1T7R | SmPLK1 | 5’-TAATACGACTCACTATAGGGCGGATGCTTTGACAGAGTGAACA-3’ (nt 1307-1329) |
| SmPLK2T7F | SmPLK1 | 5’- TAATACGACTCACTATAGGGGTTGTGTGATAACTCATATGGTGTCG-3’ (nt 1200-1225) |
| SmPLK2T7R | SmPLK1 | 5’-TAATACGACTCACTATAGGGGGAATGAGACGCTCTATCATTTC-3’ (nt 1675-1697) |
| SmSAK1T7F | SmSAK | 5’-TAATACGACTCACTATAGGGCCACCATCTTCGCAAAGTGAAGTG-3’ (nt 915-938) |
| SmSAK1T7R | SmSAK | 5’-TAATACGACTCACTATAGGGTAGATCAAGATATTTTGTCCAG-3’ (nt 1616-1637) |
| SmSAK2T7F | SmSAK | 5’-TAATACGACTCACTATAGGGGCATGCCGAGATCATTGCCG-3’ (nt 2122-2141) |
| SmSAK2T7R | SmSAK | 5’-TAATACGACTCACTATAGGGGGTAGTTCCTGATCGGTTTCAGG-3’ (nt 2572-2594) |
| mCherryT7F | mCherry | 5′-TAATACGACTCACTATAGGGATGGTGAGCAAGGGCGAGGAG-3′ (nt 1-21) |
| mCherryT7R | mCherry | 5′-TAATACGACTCACTATAGGGTTACTTGTACAGCTCGTCC-3’ (nt 693-711) |
| **Control primers used for gene expression analysis using RT-qPCR** | | |
| SmPLK1qF | SmPLK1 | 5’-CTTTGTAACCGCAGATCGCTT-3’ (nt 310-330) |
| SmPLK1qR | SmPLK1 | 5’-TGACAGCCATGGACAATTTGTT-3’ (nt 389-410) |
| SmSAKqF | SmSAK | 5’-TCCCCGGAAGTCGCTAGTC-3’ (nt 550-561) |
| SmSAKqR | SmSAK | 5’-ATCGAATGGTGGATGACCAAC-3’ (nt 631-651) |
| SmCoxqF | SmCox | 5′-TACGGTTGGTGGTGTCACAG-3′ (nt 1317-1336) |
| SmCoxqR | SmCox | 5′-ACGGCCATCACCATACTAGC-3′ (nt 1449-1468) |
| SmCB1qF | SmCB1 | 5′-ACTTGGTGGGCACGCTATAC-3′ (nt 849-868) |
| SmCB1qR | SmCB1 | 5’-GGCTGTTACCTCGGATTCAA-3′ (nt 989-1008) |

^a^ all dsRNAs target the Polo-box domain as it is unique to the Polo-kinase family. The T7 DNA polymerase binding motif is underlined**.**
