# Supplementary figures and images for: Structure-Bioactivity Relationship for Benzimidazole Thiophene Inhibitors of Polo-Like Kinase 1 (PLK1), a Potential Drug Target in Schistosoma mansoni
Source: PLoS Negl Trop Dis. 2016 Jan 11;10(1):e0004356. doi: 10.1371/journal.pntd.0004356 (PMC4709140; doi:10.1371/journal.pntd.0004356)

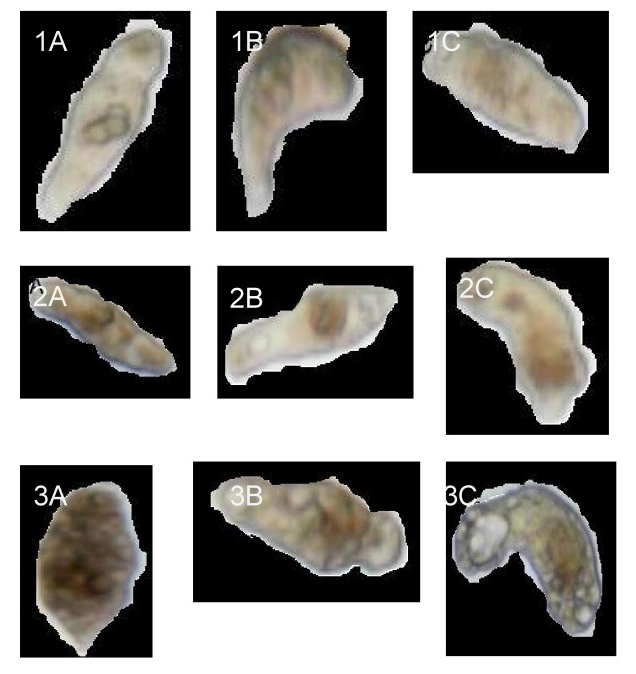

Supplement: S1 Fig — Examples of control parasites are shown in panels 1A through C. These parasites were also algorithmically grouped in a cluster that showed little or no phenotypic changes (blue in Fig 2). Panels 2A-C: examples of parasites deemed by the clustering algorithm to be unaffected by compound action (depicted in green in Fig 2). Of these, the parasites shown in Panels 2A, B and C were exposed to GSK448459A, GSK1030059A and GSK580432A, respectively. Note that even though these compounds were weakly active in terms of their effects on the parasite population, not every parasite was equally or significantly affected by the compound. Such phenotypic heterogeneity is commonly encountered as demonstrated here. Panels 3A-C: examples of parasites affected by compounds as determined both by algorithm clustering and manual scoring (represented by red points in Fig 2). The parasites shown in Panels 3A, B and C were exposed to GSK483724A, GSK641502A and GSK346294A, respectively. These compounds were also found to be active by manual scoring. (TIF) [file pntd.0004356.s004.tif]

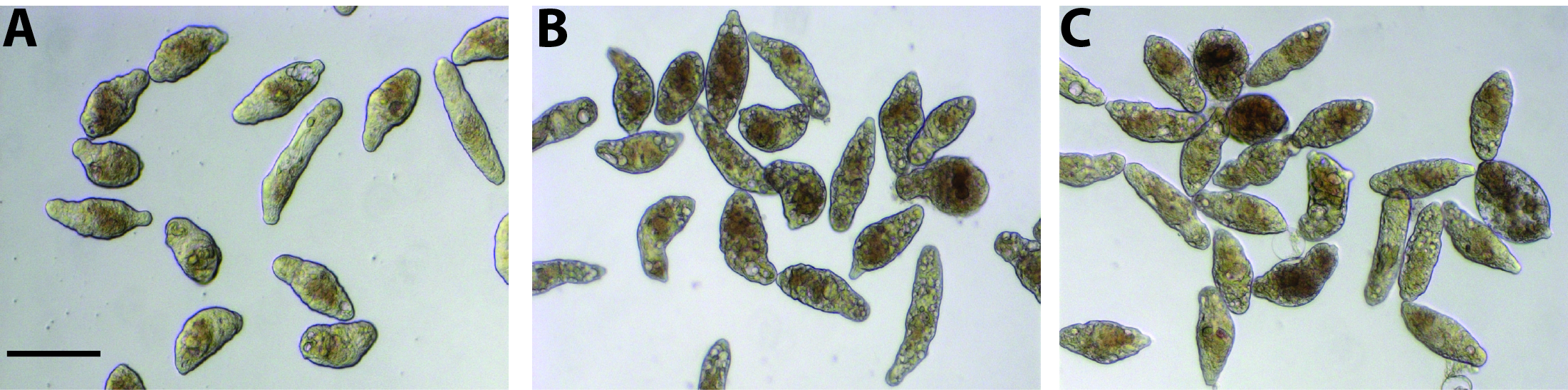

Supplement: S2 Fig — Parasites were incubated for 48 h in the presence of the appropriate DMSO control (A) or 1 μM GSK483724A (B) or GSK641502A (C). Note the internal vacuolization in parasites exposed to GSK compounds. Bar = 200 μM. (TIF) [file pntd.0004356.s005.tif]
